# Nutrition-Focused Quality Improvement Programs in Pediatric Care

**DOI:** 10.3390/children11121434

**Published:** 2024-11-26

**Authors:** Amy R. Sharn, Wendy Phillips, John T. Stutts, Mary Kaminski, Amy Shepps, Mary Beth Arensberg

**Affiliations:** 1 Global Medical Affairs and Research, Abbott Nutrition Division of Abbott, 2900 Easton Square Place, Columbus, OH 43219, USA; 2 Morrison Healthcare, 7900 Victoria Circle, Independence, OH 44131, USA; 3 Abbott Nutrition Health Institute, Abbott Nutrition Division of Abbott, 3300 Stelzer Road, Columbus, OH 43219, USA; 4 Government Affairs, Abbott, 1801 Pennsylvania Avenue, NW, Washington, DC 20006, USA; 5 Health Policy, Abbott Nutrition Division of Abbott, 3300 Stelzer Road, Columbus, OH 43219, USA

**Keywords:** pediatric malnutrition, quality improvement program, hospital care, child nutrition, food insecurity

## Abstract

Nutrition is fundamental to a child’s growth and development. However, nutritional health is often compromised by acute and chronic conditions and treatments that can commonly result in malnutrition. Malnutrition encompasses undernutrition and overnutrition and may be exacerbated by food insecurity. Recent health policy efforts in the United States (US) include those focused on quality measurement and social determinants of health (SDOH) to reduce risks for malnutrition and food insecurity. Nutrition-focused quality improvement programs (QIPs) have emerged as a successful model for benchmarking current nutrition care in adult patients and creating pathways for establishing best practices for timely malnutrition screening, intervention, and appropriate follow-up and care coordination. However, less is known about opportunities for nutrition-focused QIPs in hospital pediatric malnutrition care. This *Perspective* helps fill the gap by discussing the problem of pediatric malnutrition and current US quality frameworks and child nutrition programs related to malnutrition and food insecurity. In addition, this *Perspective* summarizes how nutrition-focused QIPs can impact malnutrition, including how QIPs can link hospital care with patient discharge planning and outpatient interventions. Finally, the *Perspective* outlines specific opportunities for the implementation of pediatric nutrition-focused QIPs to reduce office visits and/or inpatient readmissions through appropriate nutrition screening, assessment, and interventions.

## 1. Introduction

Nutrition is fundamental to a child’s growth and development. Globally, undernutrition is linked to nearly half of the deaths among children under age five [1]. The inadequate nutrient intake of energy, protein, and micronutrients crucial for child development are often the cause of growth impairment [2]. In the United States (US), such severe outcomes from undernutrition are less common. However, pediatric undernutrition still occurs in the US, often as a result of acute or chronic conditions and their treatments, as well as social determinants of health (SDOH), such as food insecurity, that can compromise children’s nutritional health. Malnutrition has diverse presentations and can include imbalances in a child’s intake of energy and/or nutrients, deficiencies, or excesses. While it can include undernutrition (micronutrient deficiencies, stunting, wasting, underweight) and overnutrition (obesity, overweight) [1], this Perspective is focused on pediatric malnutrition presenting as undernutrition in the acute care setting. 

Pediatric malnutrition can negatively impact health outcomes, but it is frequently underdiagnosed in the hospital [3,4]. Barriers can include the lack of a gold standard method and limited clinician awareness and education on malnutrition screening and assessment [5,6]. This presents an opportunity for quality improvement to educate and inform clinicians, perfect care processes, and advance performance in the US value-based healthcare system. Nutrition-focused quality improvement programs have been found to be effective in addressing hospital malnutrition in the adult population [7]. However, less is known about opportunities for nutrition-focused quality improvement programs (QIPs) in hospital pediatric malnutrition care.

This *Perspective* helps fill the gap by discussing the definition, impact, and identification of pediatric malnutrition and the current US quality frameworks and child nutrition programs related to malnutrition and food insecurity. In addition, this *Perspective* summarizes how nutrition-focused QIPs can impact malnutrition, including how QIPs can link hospital care with patient discharge planning and outpatient interventions. Finally, this *Perspective* outlines specific opportunities for the implementation of pediatric nutrition-focused QIPs to reduce office visits and/or inpatient readmissions through appropriate nutrition screening, assessment, and interventions.

## 2. Pediatric Malnutrition

Pediatric malnutrition can be defined based on its etiology, and classified as illness related, non-illness related, or a combination of both [8]. Pediatric illnesses frequently associated with malnutrition include acute conditions like trauma, burns, and infections; chronic diseases such as gastrointestinal diseases, congenital heart disease, neuromuscular diseases, cystic fibrosis, chronic kidney disease, and malignancies [8]; as well as disabilities [9]. Non-illness related factors for malnutrition can include behavioral disorders, like anorexia nervosa and food aversions, or environmental factors, and SDOH, often those related to socioeconomic conditions [8]. Food insecurity is one such socioeconomic condition and in 2023, nearly 18% of US households with children experienced food insecurity [10]. In addition, racial and ethnic disparities exist. In the US, Hispanic children can experience higher rates of stunting compared to their peers [11], and there are disparities in malnutrition among hospitalized patients in the pediatric intensive care unit (PICU) [12]. Healthcare disparities can also persist when socioeconomic discrimination and/or ethnic biases lead to less diagnoses of malnutrition [4].

The impact of pediatric malnutrition can be significant in the acute care setting, and has been associated with greater rates of infection, a longer length of hospital stay, poorer clinical outcomes [13,14,15,16], and increased healthcare costs [13]. The diagnosis of pediatric malnutrition in US hospitals has increased in the last two decades, from an overall prevalence for coded malnutrition diagnosis of 1.9% in 2002 [17] to 6.4% in 2019 [4]. In some populations, such as the PICU, coded malnutrition diagnosis is much higher; in one PICU study, malnutrition was documented at more than 20% [13].

Though evidence-based tools and best practices exist to identify and intervene for pediatric malnutrition, it remains underdiagnosed in US pediatric hospital patients [3,4] and the nutrition status of ill children often deteriorates during hospitalization [5]. A consensus statement from the Academy of Nutrition and Dietetics (Academy) and the American Society for Parenteral and Enteral Nutrition (ASPEN) in 2014 recommended specific indicators for identifying and documenting pediatric malnutrition [18]. ASPEN later developed a pediatric nutrition care pathway detailing the specific steps (from hospital admission through to discharge) for malnutrition screening, diagnosis, nutrition care plan, and intervention [19]. Guidance has also been published on the elements of a pediatric nutrition-focused physical exam for malnutrition identification [20] and the use of specific malnutrition measurement tools such as the mid-upper arm circumference (MUAC) tape [21]. The Academy has published an Evidence Analysis Center systematic review of the validity and reliability of pediatric nutrition screening tools in various care settings, including for the hospital [22]. In addition, there has been an analysis to determine the gaps in both pediatric malnutrition screening and assessment tools [23] and a recognition of the need to “standardize pediatric malnutrition risk screening using validated pediatric tools and allocate resources to perform screening” [24]. When malnutrition is indicated and nutrient needs cannot be met by food alone, pediatric oral nutrition supplements (ONS) and tube feeding products are available and supported by malnutrition intervention guidelines, such as recommendations specific to pediatric oncology [25] and the ASPEN and Society of Critical Care Medicine guidelines on providing and assessing nutrition support therapy in pediatric critical care [26].

## 3. US Healthcare Quality Policies and Child Nutrition Programs Related to Pediatric Malnutrition

Increased malnutrition risk identification and intervention are fundamental to addressing pediatric malnutrition in acute care and are influenced by US healthcare quality policies and child nutrition programs. Quality measurement is a major focus of American healthcare quality policy today, due to the consideration and adoption of the value-based care model. In the US, there are more than 2300 healthcare quality measures [27]. These quality measures have traditionally targeted adult patient care, in part because the federal government is the largest payer for older adult healthcare through the US Medicare program.

In contrast, the number of quality measures specific to the pediatric population is much more limited. Quality improvement provisions that were part of the Children’s Health Insurance Program Reauthorization Act of 2009 (CHIPRA) have led to the establishment of a Child Core Set of quality measures, with mandatory reporting by states starting in 2024 [28]. The Child Core Set includes a measure on weight assessment and counseling for nutrition/physical activity, and a measure related to low-birth weight infants [29], but no measures specifically related to acute care malnutrition. However, there are over 160 voluntary quality measures that have been developed by the federal Pediatric Quality Measures Program (PQMP), one of which does address the importance of screening for nutrition status on admission to the PICU [30]. This measure is the *Initial Baseline Screen of Nutrition Status for Every Patient Within 24 Hours of Pediatric Intensive Care Unit (PICU) Admission* [31]. A fact sheet for the PICU nutrition screening measure states, “an initial baseline screen of nutritional status for every PICU patient increases awareness of his/her nutritional state, identifies patients at risk for malnutrition and allows providers to adjust the timing, content, and quality of nutrition therapy to meet the individual patient’s needs” [32]. Increased awareness of the nutritional state could also help improve the documented diagnosis of and intervention for pediatric malnutrition, underscoring the importance of greater implementation of and reporting on this PICU measure in the acute care setting. However, there is no record in the literature of the current adoption rate for this quality measure. 

Another way to improve documented pediatric malnutrition diagnoses and interventions is to increase understanding of the non-illness related factors like SDOH that are associated with malnutrition. As part of the US Healthy People 2030 goals, access to nutritious food (food security) was identified as a SDOH [33], and nutrition/food security is known to be related to malnutrition [34]. At the community level, nutrition security has become a heightened area of focus for clinicians “in recognition of rapid increase in the prevalence of several diet-related diseases and long-standing racial disparities in access to nutritional foods and diet-related conditions” [35]. The focus is further amplified with chronic diseases beginning earlier in life; as an example, nearly one in four adolescents in the US have malnutrition presenting as overnutrition (obesity) [36].

Synergizing human services with health services (e.g., public health with medical care) is a critical step to addressing SDOH and improving equitable outcomes [37]. Similarly, the World Health Organization defines SDOH as “the conditions in which people are born, grow, work, live, and age, and the wider set of forces and systems shaping the conditions of daily life”, all of which can influence health inequities [38]. The United States Department of Agriculture (USDA) has long supported community child nutrition programs. USDA’s focus on nutrition security is evident through its Food and Nutrition Services (FNS) programs to help reduce child hunger and obesity [39], and participation in these programs has been found to reduce food insecurity and improve diet quality [40]. Table 1 shows the FNS nutrition programs’ ability to provide access to nutritious foods throughout the year, with special attention to summer breaks when school lunch is not provided to children.

With SDOH in mind, the International Classification of Disease (ICD) in 2016 added codes for SDOH, termed Z codes. These include codes for social risk factors, social needs, and adverse childhood experiences [55], as well as a specific code for food insecurity, Z59.41 [56]. Z codes that capture health-related outcomes are increasing in use, however, consistent documentation will improve non-medical factors affecting health and the healthcare team’s ability to apply appropriate interventions [57]. The Center for Medicare and Medicaid Innovation (CMMI) reports SDOH can help CMMI models achieve improved outcomes for patients, reduced healthcare spending, and an increase in overall healthier communities [58]. There has been a rise in the number of US hospitals using SDOH codes, increasing by almost 8% from 2016 to 2019 [55]. Yet the overall documentation rate of SDOH codes remains low (<2%) despite policy changes that have allowed nonphysician clinicians to code for SDOH. Possible barriers for using these codes may include a lack of SDOH ICD-10 code awareness, due to the relative newness of the code availability, and confusion about who can document for SDOH. Additionally, SDOH claims are not typically part of the healthcare payment process and therefore receive less focus [55]. Further, while the Centers for Medicare and Medicaid Services (CMS) has two new required SDOH acute care quality measures for adults [59], there are no SDOH quality measures specific to pediatric acute care. The lack of usage of the SDOH codes may lead to lower intervention rates to address issues like food insecurity and make it difficult to understand the true prevalence of food insecurity in pediatric hospitalized patients.

Usually, hospital pediatric malnutrition–whether illness related, non-illness related, or a combination of both–cannot be fully resolved during an acute care stay. Nutrition interventions starting in the hospital often need to continue post-discharge. This underscores the need for acute care institutions to work closely with community nutrition service providers. There are several ways that US healthcare quality policies and child nutrition programs offer a supportive framework and resources. For example, the Biden administration of the US has laid out a *National Strategy on Hunger, Nutrition, and Health*, which includes a pillar specifically focused on nutrition and health, and the recommended action of incentivizing payors and providers to screen for food insecurity and other SDOH [60]. In alignment with this, CMS has added the Implement Food Insecurity and Nutrition Risk Identification and Treatment Protocols Improvement Activity to several value-based care programs. This food insecurity and nutrition risk improvement activity lists specific requirements for implementing screening protocols and documenting an intervention plan for those patients identified with/at risk for food insecurity and/or malnutrition. Suggested interventions include information on Supplemental Nutrition Assistance (SNAP) enrollment, referrals for community resources such as food pantries, and follow-up calls to those families receiving such resources [61].

Similar to the acute care setting, there is underdiagnosis of pediatric malnutrition in the ambulatory care setting [62]. Implementation of the food insecurity and nutrition risk improvement activity could help address this situation and benefit acute care, as malnutrition in children with chronic diseases increases their hospital care needs upon admission [63]. For example, it is well documented that malnutrition and food insecurity are prevalent among cancer patients [64,65], and malnutrition is associated with poorer outcomes, higher emergency department utilization, and overall costs, among other negative impacts [66]. The identification of malnutrition risk and food insecurity across outpatient settings can help facilitate access to appropriate and timely nutrition interventions as well as connect patients to community-based nutrition programs and services that address ongoing needs. Nutrition intervention for malnutrition has been associated with improved health status across healthcare systems [5]. Early nutrition intervention can decrease malnutrition complication rates, costs of care, and reduce readmissions [13].

## 4. Healthcare Quality Improvement and Nutrition-Focused Quality Improvement Programs (QIPs)

As this *Perspective* has discussed, pediatric malnutrition is underdiagnosed in acute care institutions, even as there are evidence-based tools and best practices along with US-developed quality frameworks and child nutrition programs that can provide support to better identify and intervene for pediatric malnutrition. One way to build on these resources is to leverage the process of healthcare quality improvement. Among adult patients, nutrition-focused QIPs have noted improvements in identifying and managing patients at risk for/with malnutrition, in addition to significant improvements in health and economic outcomes among different patient populations (e.g., surgical, medical, diabetes, cardiovascular, and others) [67,68,69,70,71,72].

QIP studies, in general, are used to improve care systematically by standardizing processes and care structures; the goal is to reduce variation, achieve predictable results, and improve outcomes for patients and healthcare systems. Adult nutrition-focused QIPs have focused on simple steps including malnutrition screening, nutrition education, and counseling among patients identified as at risk for/with malnutrition, prompt initiation of a disease-specific ONS, re-education at discharge, and follow-up [67,68,69,70,71,72].

In adult patients, ONS use has been identified as the single most effective intervention in addressing nutrition deficiencies in individuals with malnutrition or its risk, and has led to multiple improved outcomes (including a reduced length of hospital stay, 30-day readmissions, and episode cost) in hospitalized patients [73]. A growing body of evidence has also demonstrated community and outpatient nutrition interventions, including ONS, to be effective in the prevention and treatment of malnutrition or its risk [68,74]. Similarly for pediatric undernutrition, ONS, as a nutrition intervention strategy compared to control interventions (usual diet, placebo, or dietary counselling), has been documented to have significantly better growth outcomes [75]. Another study in children with or at risk of undernutrition, documented to add ONS to dietary counselling, improved growth in weight and height, linear catch-up growth, and health outcomes. Yet, unfortunately, when compared to the strong scientific evidence supporting adult nutrition-focused QIPs and ONS [73,76,77,78,79,80,81], there is a dearth of scientific literature for pediatric care. The majority of pediatric nutrition-focused QIP studies address obesity [82,83,84,85] and the intensive care unit [86] with limited focus on acute care [87,88] and outpatient care [82,83,85], even as the studies document meaningful improvements in nutrition care.

Historically, the global pediatric malnutrition focus has been on malnutrition presenting as undernutrition in children below the age of five, especially among children less than a year of age [89]. Similar to the global malnutrition focus, US hospital coded diagnoses for malnutrition are highest among children less than one year of age, whereas the lowest rates are among children five years of age or older [17]. This trend reflects pediatric hospital admissions, where children less than four years of age have the highest admission rates, with the overall greatest number of admissions among infants less than one year old [17].

To work towards a future where pediatric malnutrition is readily identified, intervened for, and reduced, implementing nutrition-focused QIPs is an imperative interprofessional team effort to improve patients’ nutrition status, and subsequent health outcomes and healthcare costs. A recent example of this in the adult population is the Malnutrition Quality Improvement Initiative (MQii) Learning Collaborative [90], engaging 38 states and Puerto Rico, and 327 hospitals (as of 2022) to improve the nutrition care of adult acute care patients. Through the MQii, a Global Malnutrition Composite Score (GMCS) [91] was developed as the first electronic clinical quality measure for nutrition and adopted by CMS for implementation in its Hospital Inpatient Prospective Payment System program [91]. 

The MQii also developed an online toolkit of evidence-based best practices and resources for use by all members of the care team to support malnutrition quality improvement in healthcare institutions [92]. An updated version of the MQii toolkit was recently released to leverage best practice developments in clinical malnutrition quality care for adults [93]. In 2021, clinicians published a paper detailing how the MQii toolkit provided a framework for pediatric malnutrition quality improvement [94]. The MQii toolkit reflects the basic nutrition care process—which is the same whether for adult or pediatric patients [94]—although the specific malnutrition risk screening tools and indicators may differ between the populations as pediatric malnutrition care must also reflect attention to child growth and development. Further, there has been a call for a scale-out [95] of the MQii framework [96] to better fit the pediatric population, especially given the challenges of using standardized nutrition screening tools across different pediatric age groups. The Academy’s Evidence Analysis Library [97] provides scientific support for the appropriate use of nutrition screening tools among various age groups and disease states. 

**Figure 1:**
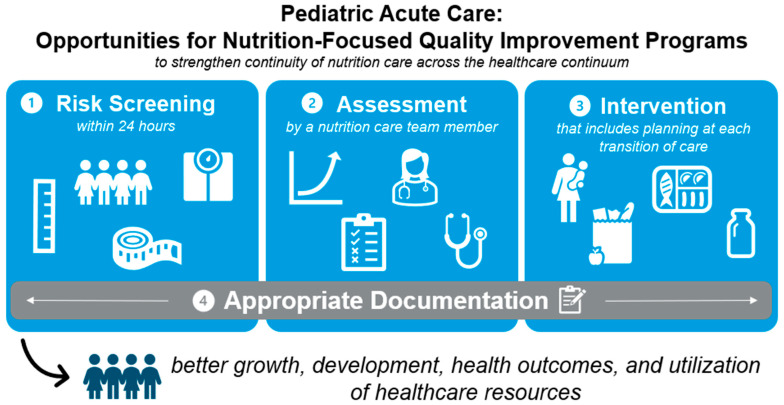
Areas for nutrition quality improvement in the pediatric acute care setting.

There remains a dire need for further work focusing on the pediatric acute care setting and the role of regular nutrition screening, intervention, and follow-up as a part of standard care processes. The MQii framework [96] offers a pathway to evaluate nutrition care in the acute setting, and includes using a readiness questionnaire to assess a facility’s alacrity to implement and create a nutrition-focused QIP. Following the readiness assessment, seeking leadership support and empowering project champions to lead the QIP are crucial to ensure its success [96]. Per the recommendations, the QIP’s focus should be these four areas of improvement [96]:

(1) Risk screening—nutrition risk screening should occur within 24 h of admission;
(2) Assessment—an assessment for nutrition risk should be completed by a nutrition care team member;
(3) Intervention—nutrition intervention should also include discharge planning as a best practice to reduce the likelihood of readmissions;
(4) Appropriate Documentation—documentation should occur at each stage of the QIP, with special attention to the malnutrition diagnosis to ensure appropriate action (Figure 1).

In implementing these steps using the MQii framework [96], future nutrition-focused QIPs should be considered and developed to scale-out (the transfer of methods to a new population or setting) [95] to the outpatient space. This would strengthen the continuity of nutrition care across the healthcare continuum, leading to better health outcomes and the utilization of healthcare resources.

## 5. Summary

Pediatric malnutrition impacts a child’s growth, development, and healthcare outcomes, and while diagnosis rates have improved, malnutrition continues to be underdiagnosed in US hospitals. A US PICU quality measure and an outpatient improvement activity support improved malnutrition identification and intervention. However, there is limited data regarding their uptake, and more research is needed to better understand the impact of nutrition interventions on improving patient health and clinical and economic outcomes. To address the care gap in pediatric malnutrition care in the hospital, nutrition care needs to be prioritized, and the nutrition education and training of hospital staff needs to improve. Nutrition-focused QIPs provide real-world evidence that pediatric malnutrition diagnosis and intervention can be improved. Frameworks like the MQii toolkit can be adapted to guide nutrition-focused QIP development and implementation to benefit pediatric care and health outcomes.

## Figures and Tables

**Table 1:** The United States Department of Agriculture (USDA) Food and Nutrition Services’ programs supporting child nutrition.

Nutrition Assistance Programs	
Farmers Market Nutrition Program (FMNP)	FMNP is a program for WIC participants to receive fresh and locally grown produce; it expands the awareness and use of, as well as sales at, farmers markets [41].
Supplemental Nutrition Assistance Program (SNAP)	SNAP supports families with low income through food benefits that supplement their grocery budget [42].
Special Supplemental Nutrition Program for Women, Infants, and Children (WIC)	WIC is for low-income infants; children up to age five; and pregnant, breastfeeding, and non-breastfeeding post-partum women at nutrition risk, and provides healthcare referrals, nutrition education, and benefits for specific supplemental foods [43].
Child Nutrition Programs	
Child and Adult Care Food Program (CACFP)	CACFP reimburses participating emergency shelters, afterschool care programs, home daycare programs, and childcare centers for nutritious meals and snacks provided to eligible children [44].
Fresh Fruit and Vegetable Program (FFVP)	FFVP provides children at eligible elementary schools with a variety of free, fresh fruit and vegetable snacks [45].
National School Lunch Program (NSLP)	NSLP provides funding for nutritionally balanced, low-cost, or free lunches to participating residential childcare institutions and public and nonprofit private schools [46].
School Breakfast Program (SBP)	SBP reimburses states for implementing nonprofit breakfast programs in residential childcare institutions and schools [47].
Special Milk Program (SMP)	SMP is for children attending schools and childcare institutions that do not participate in other federal meal service programs; SMP provides milk [48].
SUN Bucks (Summer Electronic Benefits Transfer (EBT))	SUN Bucks is a summer grocery benefit for eligible school-aged children; families can receive SUN Bucks in addition to SNAP and WIC benefits [49].
SUN Meals (Summer Food Service Program (SFSP))	SUN Meals are provided free in the summer at participating schools, parks, and other neighborhood locations to eligible school-aged children [50].
The Patrick Leahy Farm to School Program	The Patrick Leahy Farm to School Program works with tribal communities to support child nutrition programs by incorporating local foods [51].
USDA Food Distribution Programs	
Food Distribution Program on Indian Reservations (FDPIR)	FDPIR is for income-eligible families on Native American reservations and Native American families living near reservations; it provides USDA Foods [52].
The Emergency Food Assistance Program (TEFAP)	TEFAP supports low-income families with free emergency food assistance [53].
USDA Foods in Schools	The USDA Foods in Schools program provides 100% American-grown and produced foods to schools, child care facilities, and institutions participating in NSLP, CACFP, SFSP, and other child nutrition programs [54].

CACFP = Child and Adult Care Food Program; FDPIR = Food Distribution Program on Indian Reservations; FFVP = Fresh Fruit and Vegetable Program; FMNP = Farmers Market Nutrition Program; NSLP = National School Lunch Program; SBP = School Breakfast Program; SMP = Special Milk Program; SNAP = Supplemental Nutrition Assistance Program; SUN Bucks = Summer Electronic Benefits Transfer (EBT); SUN Meals = Summer Food Service Program (SFSP); TEFAP = The Emergency Food Assistance Program; USDA = United States Department of Agriculture; WIC = Special Supplemental Nutrition Program for Women, Infants, and Children.
